# Serological and molecular inquiry of Chagas disease in an Afro-descendant settlement in Mato Grosso do Sul State, Brazil

**DOI:** 10.1371/journal.pone.0189448

**Published:** 2018-01-09

**Authors:** Mariana Furquim da Silva Martins, Mariane Barroso Pereira, Juliana de Jesus Guimarães Ferreira, Adriana de Oliveira França, Marlon Cézar Cominetti, Eduardo de Castro Ferreira, Maria Elizabeth Moraes Cavalheiros Dorval, Cláudio Lúcio Rossi, Sílvia de Barros Mazon, Eros Antonio de Almeida, Sandra Cecília Botelho Costa, Gláucia Elisete Barbosa Marcon

**Affiliations:** 1 Departamento de Clínica Médica, Faculdade de Ciências Médicas, Universidade Estadual de Campinas (UNICAMP), Campinas, São Paulo, Brazil; 2 Centro de Ciências Biológicas e da Saúde, Faculdade de Farmácia, Universidade Federal de Mato Grosso do Sul (UFMS), Campo Grande, Mato Grosso do Sul, Brazil; 3 Universidade Anhanguera UNIDERP, Campo Grande, Mato Grosso do Sul, Brazil; 4 FIOCRUZ Mato Grosso do Sul, Fundação Oswaldo Cruz, Campo Grande, Mato Grosso do Sul, Brazil; 5 Departamento de Patologia Clínica, Faculdade de Ciências Médicas, Universidade Estadual de Campinas (UNICAMP), Campinas, São Paulo, Brazil; US Food and Drug Administration, UNITED STATES

## Abstract

Furnas do Dionísio is a Brazilian Afro-descendant settlement in the city of Jaraguari, 21.4 miles from Campo Grande, Mato Grosso do Sul, Brazil. Approximately 96 families live in this *quilombola* (Maroon) settlement, also known in Brazil as a remnant community of descendants of African slaves. Recent studies found 20% of households were infested by triatomines, 18% of insects captured in the community were infected by *Trypanosoma cruzi*, and 22.7% of dogs presented *T*. *cruzi* antibodies. The low prevalence of Chagas disease observed in humans in Mato Grosso do Sul State is attributed to its arrival via colonist migration and subsequent transplacental transmission. In order to gain a better understanding of the *T*. *cruzi* cycle in residents of the study community, serological and molecular tests were carried out to diagnose Chagas disease. In the present study, 175 residents between 2 and 80 years old were included. A total of 175 participants were interviewed and 170 provided blood samples, which were tested for *T*. *cruzi* antibodies with serological tests. Molecular diagnosis was performed in 167 participants by PCR (KDNA) and NPCR (satellite DNA) tests. One of the 170 samples tested positive for all serological tests performed. The overall frequency of Chagas disease in the community was low (0.6%). Interview responses revealed that 66.3% knew of triatomine insects and 65.7% reported having had no contact with them. Physical improvements to residences, together with vector surveillance and control by the State and municipal governments and local ecological conservation contribute to the low frequency of the Chagas disease in this *quilombola* community.

## Introduction

Furnas do Dionísio is a community predominantly comprised of descendants of African slaves (*quilombolas* or Maroons) in the city of Jaraguari, located 21.4 miles from Campo Grande, the capital of Mato Grosso do Sul State, Brazil. This community remained isolated until several decades ago due to difficult access and cultural differences. However, the community’s social customs and values were preserved. There are approximately 96 households in the settlement and more than 90% of residents are Afro-descendants. The main chronic diseases in Furnas do Dionísio are sickle cell anemia and hypertension. In 1997, the National Health Foundation (FUNASA) started a project to build or rebuild homes in the community to eradicate Chagas disease and improve the quality of life of residents [1]. Significant improvements in sanitary, educational, and social conditions occurred between 2010 and 2013. Only *quilombola* communities recognized by the federal government, of which there are 2,197 [2], participate in these actions.

Chagas disease in Brazil [3] and in Mato Grosso do Sul State receive insufficient research attention. *Triatoma infestans*, the main transmitting insect of Chagas disease is not common in the Brazilian cerrado and Pantanal. Certification of interruption of transmission by *Triatoma infestans* occurred in 2006. However, *Triatoma sordida* is a widespread species in Mato Grosso do Sul habitats, is frequent in peridomicile environments, and has the capacity to establish itself in house interiors, especially in homes presenting precarious living conditions and physical characteristics [4].

*T*. *sordida* is native to the Brazilian cerrado, mainly located in the Central-West region, and has the capacity to establish itself inside houses when natural habitats are destroyed, or food sources are scarce [5, 6].

Research carried out in Mato Grosso do Sul State showed intense presence of *T*. *sordida* around and inside houses. The occurrence of *Trypanosoma cruzi* in this predominant insect species was 0.5% inside households [4]. In Furnas do Dionísio, Cominetti and collaborators [7] found 20% of houses were infested by triatomines. Parasitological and molecular techniques demonstrated the presence of *T*. *cruzi* in 18% of captured insects.

In Furnas do Dionísio, immunological tests demonstrated the presence of *T*. *cruzi* antibodies in 22.7% of dogs, corroborating the understanding that dogs are reservoirs for *T*. *cruzi* and play an important role in its domestic and wild cycles. This study was important for determining the presence of trypanosomatids in this community and the risk to humans of contracting the disease [8]. Locally contracted human cases of Chagas disease have also been reported in Mato Grosso do Sul State [9, 10].

*Trypanosoma cruzi* and *Leishmania* spp. belong to order the Kinetoplastida and family Trypanosomatidade. Serological tests for Chagas disease may show cross reaction with Leishmaniasis. In Mato Grosso do Sul State, leishmaniasis is endemic and considered a significant public health problem, with 42.9 cases of visceral leishmaniasis and 21.6 of cutaneous leishmaniasis per 100,000 inhabitants being reported from 2010 to 2013 [11].

Investigations in Mato Grosso do Sul State demonstrated low prevalence of Chagas disease cases (0.9%), which is related to the state’s history of colonization, mainly through migration from the South and Southeast regions, where Chagas disease is endemic. Local infections may occur through transplacental transmission [9, 10]. Therefore, studies of the presence of *T*. *cruzi* are needed to establish the epidemiological profile of Chagas disease in Mato Grosso do Sul State.

The aim of this study is to determine the frequency of Chagas disease and evaluate possible associations with epidemiological and social factors in a rural population of *quilombolas* in Mato Grosso do Sul.

## Material and methods

Furnas do Dionísio is an Afro-descendant settlement located in the city of Jaraguari, 21.4 miles from Campo Grande, the capital of Mato Grosso do Sul State. The community occupies approximately 1,031 ha, with its center at the coordinates 20°9′1.34″S and 54°34′27.17″W. The community is comprised of small rural properties with masonry houses [7].

### Ethics committee

The present study was approved by the Ethics Committee of the Federal University of Mato Grosso do Sul under the CAAE number: 16382413.6.0000.0021. Individuals over 18 years signed the consent form and responded to the social and epidemiological questionnaire. Parents of children and youth under 18 years provided written consent for collection of blood and interview data.

### Socio-epidemiological interview and sample collection

A total of 175 patients were screened for Chagas disease from July to December 2014 (S1 Table). The social and epidemiological questionnaire included questions regarding personal information, such age, sex, and educational level, and epidemiological data, such as contact with triatomines, knowledge of Chagas disease, and clinical signs such as fatigue, dysphagia, and/or difficulty with evacuation. Interviews were conducted and blood samples collected by members of the research team during the same opportunity (S1 Questionnaire). For children under six, a parent or legal guardian provided responses. An anonymous identification number was used to identify information in questionnaires. A total of 175 blood samples were collected for molecular and serological tests.

### Serological tests

One hundred and seventy participants were evaluated with serological tests. *T*. *cruzi* IgG antibodies were measured by indirect immunofluorescence (IIF), chemiluminescent microparticle immunoassay (CMIA), and enzyme-linked immunosorbent assay (ELISA). IIF tests were performed using *T*. *cruzi* epimatigotes as antigen (Imunocruzi^®^, Biolab-Mérieux, Rio de Janeiro, Brazil) and an anti-human IgG fluorescein conjugate (Fluoline G^®^, bioMérieux, Lyon, France). IIF titers ≥ 1:40 were considered reactive (R) for *T*. *cruzi* antibodies. CMIA tests were performed on fully automated equipment (Abbott Architect i2000SR) using the Architect Chagas Assay (Abbott Laboratories, Germany), according to the manufacturer's instructions. Assay results are presented as a ratio of the specimen signal in relative light units (RLUs) to the cutoff value (S/CO), where S/CO values < 0.8 are considered nonreactive (NR) for *T*. *cruzi* antibodies, S/CO values ≥ 1.00 are considered reactive (R) for *T*. *cruzi* antibodies, and S/CO values from ≥ 0.80 to < 1.00 are considered grayzone (GZ). All samples were tested by ELISA (Dia Pro Diagnostic Bioprobes *T*. *cruzi*–Ab), following the manufacturer’s instructions and standard laboratory protocols.

### Molecular tests

#### DNA isolation

DNA from whole blood samples was extracted in 175 samples, using the Wizard® Genomic DNA purification kit Promega, following the manufacturer's instructions. DNA quality and absence of inhibitors were tested with amplification of the human β- globin gene using Go Taq® Green Master Mix, 1 μl of DNA in a final volume for 20 μL. PCR for *β-globin* using PCO3: 5′ ACACAACTGTGTTCACTAGC 3′ and PCO4: 5′ CAACTTCATCCACGTTCACC 3′ primers under the following PCR conditions: initial denaturation at 95°C for 15 min, 35 cycles with the cycling profile of 95°C for 1 min, 52°C for 1 min, 72°C for 1 min, and final extension for 5 min at 72°C.

#### PCR and NPCR

Detection of *T*. *cruzi* parasites kDNA (kinetoplast DNA) PCR was performed using the primers kDNA, S35: 5’AAATAATG (T/G) ACGGGTGAGATG CA 3’ and S36: 5’ GGGTTCGATTGGGGTTGGTGT 3’ generating a fragment of 330bp [12]. The PCR reaction was prepared to a final volume of 20 μL using 1 μL of DNA template, 50 mM of potassium chloride, 10 mM of Tris-HCl (pH 8.4), 2.0 mM MgCl_2_, 2.0 mM dNTP mix (Ludwig Biotec), 5.0 units of Platinum Taq polymerase (Invitrogen) and 10 pmol of each primer. Amplification of template DNA was processed in an automatic thermocycler (MJ Research PTC 200 Thermal Cycler) with initial denaturation at 94°C for 5 min, alternating 30 cycles of denaturation at 94°C for 1 min, annealing at 65°C for 1 min and extension at 72°C for 1min, followed by a step of final extension at 72°C for 10 min.

Another target for amplification by Nested-PCR (NPCR), directed to satellite DNA, was performed utilizing the primers TCZ1: 5’ CCGAGCTCTTGCCCACACGGGTGCT 3’; 5’ TCZ2: CCTCCAAGCAGCGGATAGTTCAGG3’ for first reaction and TCZ3: 5’ TGCTGCA(G/C)TCGGCTGATCGTTTTCGA 3’; TCZ4: 5’ CA(A/G)G(C/G)TTGTTTGGTGTCCAGTGTTGTGA 3’ for second reaction, [13, 14]. The first reaction was performed with 1 μL of the DNA sample, 50 mM of potassium chloride, 10 mM of Tris-HCl (pH 8.4), 1.8 mM magnesium chloride, 10 pmol of each oligonucleotide (TCZ1/TCZ2), 1.5 mM dNTP mix (Ludwig Biotec) and 5.0 U of Platinum *Taq* DNA polymerase (Invitrogen) for a final volume of 20 μL. The second reaction was performed with TCZ3 and TCZ4 primers following the same protocol. For the second reaction, the MgCl_2_ concentration was modified to 2.0 mM and a sample of the PCR product (0.5–1.0 μL) was re-amplified with the TCZ3 and TCZ4 oligonucleotides, which resulted in a fragment of 149 bp for positive samples [14].

The temperature profile for denaturation was 95°C for 5 min. The reaction mixtures were subjected to 5 cycles of amplification in a programmable thermal cycler. During each cycle, the samples were incubated at 95°C for 30 s, 60°C for 30 s and 72°C for 1 min. In the next 25 cycles, the anneal temperature of primers was altered to 65°C. At the end of the last cycle, samples were incubated at 72°C for 10 min. Approximately 0.7 μL of the reaction was used for the second amplification, where primers TCZ3 and TCZ4 amplified a 149 nucleotide internal sequence of the same repetitive sequence. In the NPCR, the cycles were differentiated: 94°C for 5 min (one cycle); 94°C for 40 s; 55°C for 40 s; 72°C for 1 min and 30 s (25 cycles); and a final extension for seven minutes (72°C). The amplified fragment of 149 bp was separated by electrophoresis on a 2% agarose gel stained with ethidium bromide and visualized by ultraviolet transilluminator.

Positive controls for PCR assays were isolated a patient with clinical compatibility, positive serology, and positive epidemiology for Chagas disease. Blood samples were multiplied by blood culture and genotyped as DTU II. As a negative control, DNA of individuals with clinical, serological, and epidemiology negatives for Chagas disease were included. To check the quality of reagents, all reagents without DNA in each reaction were subjected to amplification cycles.

## Results

Questionnaire data were tabulated and simple percentages were calculated for each item. A total of 175 participants and/or parents or guardians agreed to be interviewed (Table 1). Of the 16 children (9.1%) under six years old who participated in the study, complete information was collected for 5 (31.3%). Of the 39 (22.3%) participants in the age group 7 to 14 years, incomplete information was registered for 12 (30.7%). Of the 175 participants, interview data for 23 (13.1%) children aged 2 to 14 years old were incomplete or absent. The data in Table 1 shows the age groups of interviewees (2–6 and 7–14 years). Table 2 shows responses to questions regarding epidemiological and social conditions. The proportions of missing data due to lack of response varied between questions from 10.8% to 14.8%, due to incomplete or absent answers in the age group 2 to 14 years.

**Table 1:** Socioeconomics and epidemiological variables in the study population (n = 175).

Variable	n (%)
**Sex**	
male	96 (45.1)
female	79 (54.9)
**Age**	
≤ 6	16 (9.1)
7–14	39 (22.3)
15–39	49 (28)
40–59	58 (33.1)
≥ 60	13 (7.5)
**Mean ± SD**	**31.7 ± 21**
**Birth location**	
*Quilombola* community (MS)	60 (34.3)
City of Campo Grande (MS)	97 (55.4)
Other places	18 (10.3)
**Educational level**	
None	23 (13.1)
Primary school	117 (66.9)
Secondary school	34 (19.4)
Higher education	01 (0.6)

SD: standard deviation

**Table 2:** Participants’ knowledge of Chagas disease (n = 75).

Variable	n (%)
**Knowledge about Chagas disease**	
No	87 (49.7)
Yes	65 (37.15)
N/A	23 (13.15)
**Family history**	
No	127 (72.6)
Yes	22 (12.6)
N/A	26 (14.8)
**Knowledge of the vector**	
No	33 (18.9)
Yes	116 (66.3)
N/A	26 (14.8)
**Contact with vector**	
No	115 (65.7)
Yes	36 (20.6)
N/A	24 (13.7)
**Blood transfusion**	
No	146 (83.4)
Yes	7 (4)
N/A	22 (12.6)
**Previously tested for Chagas disease**	
No	124 (70.9)
Yes	31 (17.7)
N/A	20 (11.4)
**Difficulty evacuating and/or dysphagia**	
No	147 (84)
Yes	7 (4)
N/A	21 (12)
**Fatigue after slight exertion**	
No	111 (63.4)
Yes	45 (25.7)
N/A	19 (10.9)

N/A: No answer

A total of 175 participants provided biological samples. Of these, 79 (45.1%) were female and 96 (54.9%) were male. Additionally, 58 (33.1%) were from 40 to 59 years of age and 177 (66.9%) had no more than primary education (Table 1). Socio-epidemiological characteristics, including contact with triatomines, knowledge of Chagas disease, and clinical signs, are presented in Table 2. Of 175 participants, 127 (72.6%) did not have relatives with Chagas disease. Whereas 116 (66.3%) reported having knowledge the insect, 115 (65.7%) indicated having had no physical contact with it.

Serological tests were performed in 170 of the 175 samples collected, due to lack of serum in five samples. The prevalence of Chagas disease was 0.6% (01/170). Of the 170 serum samples tested with ELISA, CMIA, and IIF, only one sample (FD 75) was positive. This sample presented values for ELISA and CMIA ≥ 1.00. The IIF titers ≥ 1:40 were considered reactive (R) for *T*. *cruzi* antibodies. This sample (FD75) was from a 25 years old woman who was born and lived in the *quilombola* community.

**Fig 1:**
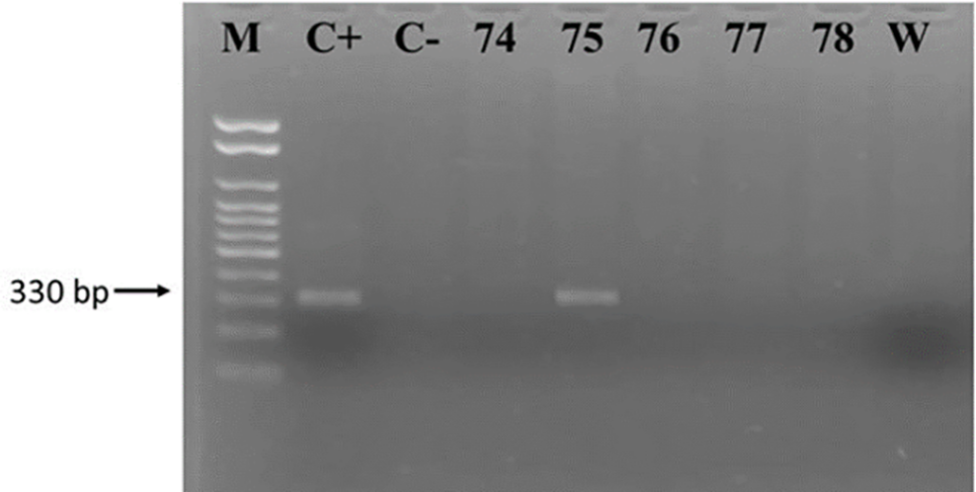
PCR results, KDNA target. M: Ladder 100 bp, Invitrogen ®; C+: *T*. *cruzi* DTU II; C-: DNA of patients who tested negative for Chagas disease; 75: PCR positive for *T*. *cruzi* KDNA; 74, 76–78: PCR negative for *T*. *cruzi* KDNA; W: PCR without DNA.

**Fig 2:**
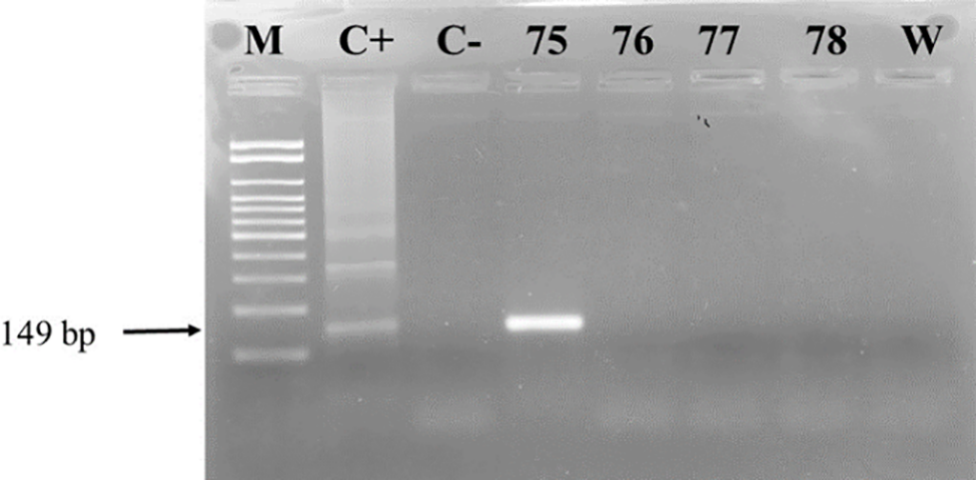
Nested PCR results, satellite DNA targets. M: Ladder 100 bp, Invitrogen ®; C+: *T*. *cruzi* DTU II; C-: DNA of patients with negative serologies and epidemiologies for Chagas disease; 75: NPCR positive for *T*. *cruzi* satellite DNA; 76–78: PCR negative for *T*. *cruzi* satellite DNA; W: NPCR without DNA.

Of the 175 samples subjected DNA extraction and subjected to the PCR test for the human β-globin gene, 167 presented DNA with adequate quality for detection of *T*. *cruzi* with PCR. These were therefore tested for the two targets KDNA (Fig 1) and satellite DNA (Fig 2). Only participant FD75 presented positive, consistent with the serological test results.

## Discussion

In the present study, the low prevalence (0.6%) of Chagas disease in an Afro-descendant settlement in Mato Grosso do Sul State was detected by molecular (PCR) and serological (IIF, ELISA, and CMIA) tests. This finding is attributable to the ecology of the primary transmitting insect (*T*. *infestans*), which is uncommon in the Brazilian cerrado and Pantanal and is considered controlled in Brazil [15, 16].

*T*. *sordida* is the most important vector species for *T*. *cruzi* in the Central-West region and is most abundant in peridomicile habitats [4]. The vector capacity of *T*. *sordida* is limited because it is mainly ornithophilic. Low rates of *T*. *cruzi* are observed in birds because they are refractory to infection. The insect is commonly established in the peridomicile and may be part of wild and domestic cycles, but has a weak tendency to invade or colonize households [16, 17, 18, 19].

Domestic mammals are considered sentinels because their infection by *T*. *cruzi* precedes human infection [20]. Chagas disease has been observed in dogs in this community [21], although the present study did not find an expressive frequency of *T*. *cruzi* infection in the human population.

Another important factor in the observed low prevalence of Chagas disease is that the study community has undergone major housing changes since 1997, including the construction or reconstruction of masonry houses by a National Health Foundation (FUNASA) project aimed to eradicate Chagas disease [1]. This intervention reduced the infestation of triatomines inside the houses and, consequently, impeded the transmission of the disease in the population.

According to the II Brazilian Consensus on Chagas Disease (II Consenso Brasileiro em Doença de Chagas) [22], laboratorial diagnosis should be performed by two serological tests based on different principles. Hemagglutination, IIF, and ELISA are the most commonly used tests. Despite advances, these serological tests can result in cross-reactions, especially where leishmaniasis is endemic. Parasitological tests such as xenodiagnosis and blood culture are quite specific, but have low sensitivity in cases of chronic Chagas disease with small numbers of circulating parasites. DNA amplification by PCR is a sensitive and specific test to detect *T*. *cruzi* DNA in blood samples obtained from chronic chagasic patients, and therefore is a promising tool to complement conventional serological tests [23].

Diagnosis by PCR should not be used as a routine test because there is no standard protocol [22, 24]. However, despite differences in sensitivity and specificity requirements between protocols [24], PCR is used as a complementary test for Chagas disease diagnosis, especially in patients with doubtful [23] or negative serology but with clinical and epidemiological signs validating obtained results [25].

In the present study, three immunological tests (IIF, CMIA, and ELISA) and two PCR targets (kDNA and nuclear DNA) had the same result, confirming the high sensitivity of serological tests and high specificity of PCR. During the chronic phase of Chagas disease, the sensitivity of ELISA and CMIA tests is 100%, while IIF reaches 93%. The specificity of these immunological tests is 98.7% to 100% for ELISA, 99% for IIF, and 97.5% for CMIA [26, 27]. Due to the lack of accuracy of commercially available serological tests, a review by Coura and colleagues [28] reinforces that two serological tests are necessary for laboratory diagnosis of chronic Chagas disease, as currently indicated by the II Brazilian Consensus on Chagas Disease [22].

Studies in Mato Grosso do Sul demonstrate that Chagas disease prevalence in humans is low (between 0.05% and 3%) and local infections are present [9, 10, 28, 29, 30, 31]. In this study, the results confirm the low prevalence (0.6%) of Chagas disease in the rural community of Furnas do Dionísio in Mato Grosso do Sul State, as previously reported in the literature.

Interview data were incomplete or absent for 23 children (13.1%) between 2 and 14 years old, representing a potential study limitation with regard to questions addressing knowledge of Chagas disease (Table 2). Because the *quilombola* settlement is located about 21.4 miles from Campo Grande, approximately half of residents (55.4%) were born in this city. Most of the adult population had some primary education and the community now has a school offering primary and secondary education, which is attended by most children and youth. The majority of participants (72.6%) reported having no family history of Chagas disease and 49.7% had no previous knowledge of it (Table 2). Yet, 66.3% reported having knowledge of the insect, which they encounter in corrals, pigsties, chicken coops, and barns (data not shown). These triatomines may or may not be relevant vectors of Chagas disease in this community. Because the local environment is rural and close to natural vegetation, other genera in the Triatominae family may also be present.

Positive aspects of the study included seeking to evaluate the entire population, which represented a wide range of ages, from 2 to 83 years and with a mean age of 31.7 ± 21 years. These findings suggest there is no vector transmission in this community despite evidence that *T*. *cruzi* is present in domestic and synanthropic animals [7] and in triatomines [32]. Furthermore, the results suggest that the parasite cycle occurs in the wild, mainly in the preserved natural areas near the community. This is the first published study of Chagas disease prevalence in a *quilombola* community in Mato Grosso do Sul, Brazil. In a rural area of Mexico, a similar study demonstrated a low seroprevalence of 4.8%, even though triatomines were abundant in the region [33]. Similar to the present study, this research in Mexico reinforces that vector surveillance with health education programmes in communities should be strengthened.

The Chagas disease is considered emergent in Texas (USA) and Europe. There is growing concern within the scientific community about the emergence and reemergence of neglected diseases [34, 35]. According to the II Brazilian Consensus on Chagas Disease [22], which estimated the overall prevalence in Brazil to be between 1% and 2.4%, more studies and inquiries should be carried out that integrate the production of knowledge of the disease between such diverse fields as epidemiology and transmission dynamics. This study in the Afro-descendant settlement Furnas do Dionísio revealed a prevalence of 0.6%, corroborating previous findings in the same community [7, 32]. Vector transmission was low and the *Trypanosoma cruzi* cycle occurs in natural habitats.

The low prevalence of Chagas disease in the Furnas do Dionísio quilombola community suggests that the *T*. *Cruzi* cycle is present and occurs between synanthrope, wild, and domestic animals. Human involvement is unlikely. While carrying out the present study, it was evident that a number of the residents customs, such as cultivating foods, raising animals, respecting the land, and concerning themselves with conserving natural vegetation around their properties, resulted in respect for the environment and sustainable production. Care for the environment observed in the community is important for controlling anthropozoonoses and other disorders that may results from lack of environmental protection.

## Supporting information

S1 Table Patient data. (DOCX)

S1 Questionnaire Social and epidemiological questionnaire for Chagas disease. (DOCX)
