# Supplementary material for: Serological and molecular inquiry of Chagas disease in an Afro-descendant settlement in Mato Grosso do Sul State, Brazil
Source: PLoS One. 2018 Jan 9;13(1):e0189448. doi: 10.1371/journal.pone.0189448 (PMC5760030; doi:10.1371/journal.pone.0189448)
Supplement: S1 Table — (DOCX) [file pone.0189448.s001.docx]

**Supporting 1** : Patients data

| Patient | Age | Gender | IIF | CMIA | ELISA | PCR KDNA | NPCR sat DNA |
| --- | --- | --- | --- | --- | --- | --- | --- |
| FD01 | 53 | M | NR | NR | NR | N | N |
| FD02 | 42 | M | NR | NR | NR | **NT** | **NT** |
| FD03 | 40 | M | NR | NR | NR | N | N |
| FD04 | 52 | M | NR | NR | NR | N | N |
| FD05 | 43 | F | NR | NR | NR | N | N |
| FD06 | 57 | M | NR | NR | NR | N | N |
| FD07 | 42 | M | NR | NR | NR | N | N |
| FD08 | 44 | F | NR | NR | NR | N | N |
| FD09 | 45 | M | NR | NR | NR | N | N |
| FD10 | 06 | F | NR | NR | NR | N | N |
| FD11 | 24 | F | NR | NR | NR | N | N |
| FD12 | 15 | F | NR | NR | NR | N | N |
| FD13 | 44 | F | NR | NR | NR | N | N |
| FD14 | 46 | F | NR | NR | NR | N | N |
| FD15 | 49 | M | NR | NR | NR | N | N |
| FD16 | 20 | F | NR | NR | NR | N | N |
| FD17 | 19 | F | NR | NR | NR | N | N |
| FD18 | 50 | F | NR | NR | NR | N | N |
| FD19 | 20 | M | NR | NR | NR | N | N |
| FD20 | 49 | F | NR | NR | NR | N | N |
| FD21 | 52 | M | NR | NR | NR | N | N |
| FD22 | 70 | F | NR | NR | NR | **NT** | **NT** |
| FD23 | 87 | F | NR | NR | NR | N | N |
| FD24 | 24 | F | NR | NR | NR | N | N |
| FD25 | 49 | F | NR | NR | NR | N | N |
| FD26 | 02 | F | **NT** | **NT** | **NT** | **NT** | **NT** |
| FD27 | 04 | M | NR | NR | NR | N | N |
| FD28 | 54 | F | NR | NR | NR | N | N |
| FD29 | 17 | F | NR | NR | NR | N | N |
| FD30 | 03 | F | **NT** | **NT** | **NT** | **NT** | **NT** |
| FD31 | 41 | F | NR | NR | NR | N | N |
| FD32 | 11 | F | NR | NR | NR | N | N |
| FD33 | 14 | M | NR | NR | NR | N | N |
| FD34 | 43 | F | NR | NR | NR | N | N |
| FD35 | 13 | F | NR | NR | NR | N | N |
| FD36 | 08 | F | NR | NR | NR | N | N |
| FD37 | 10 | F | NR | NR | NR | N | N |
| FD38 | 11 | F | NR | NR | NR | N | N |
| FD39 | 06 | F | NR | NR | NR | N | N |
| FD40 | 58 | F | NR | NR | NR | N | N |
| FD41 | 57 | M | NR | NR | NR | N | N |
| FD42 | 32 | F | NR | NR | NR | N | N |
| FD43 | 09 | F | NR | NR | NR | N | N |
| FD44 | 25 | F | NR | NR | NR | N | N |
| FD45 | 07 | M | NR | NR | NR | N | N |
| FD46 | 59 | F | NR | NR | NR | N | N |
| FD47 | 10 | M | NR | NR | NR | N | N |
| FD48 | 35 | M | NR | NR | NR | N | N |
| FD49 | 11 | M | NR | NR | NR | N | N |
| FD50 | 07 | F | NR | NR | NR | N | N |
| FD51 | 06 | F | NR | NR | NR | N | N |
| FD52 | 62 | M | NR | NR | NR | N | N |
| FD53 | 09 | M | NR | NR | NR | N | N |
| FD54 | 46 | M | NR | NR | NR | N | N |
| FD55 | 12 | F | NR | NR | NR | N | N |
| FD56 | 13 | M | NR | NR | NR | N | N |
| FD57 | 39 | F | NR | NR | NR | N | N |
| FD58 | 07 | M | NR | NR | NR | N | N |
| FD59 | 52 | M | NR | NR | NR | N | N |
| FD60 | 69 | F | NR | NR | NR | N | N |
| FD61 | 38 | F | NR | NR | NR | N | N |
| FD62 | 51 | F | NR | NR | NR | N | N |
| FD63 | 50 | F | **NT** | **NT** | **NT** | **NT** | **NT** |
| FD64 | 59 | F | NR | NR | NR | N | N |
| FD65 | 88 | F | NR | NR | NR | N | N |
| FD66 | 53 | F | NR | NR | NR | N | N |
| FD67 | 64 | M | NR | NR | NR | N | N |
| FD68 | 26 | F | NR | NR | NR | N | N |
| FD69 | 40 | M | NR | NR | NR | N | N |
| FD70 | 33 | M | NR | NR | NR | N | N |
| FD71 | 44 | F | NR | NR | NR | N | N |
| FD72 | 69 | M | NR | NR | NR | N | N |
| FD73 | 12 | M | NR | NR | NR | N | N |
| FD74 | 49 | F | NR | NR | NR | N | N |
| FD75 | **23** | **F** | **R** | **R** | **R** | **P** | **P** |
| FD76 | 41 | F | NR | NR | NR | N | N |
| FD77 | 05 | F | NR | NR | NR | N | N |
| FD78 | 33 | M | NR | NR | NR | N | N |
| FD79 | 11 | F | NR | NR | NR | N | N |
| FD80 | 80 | M | NR | NR | NR | N | N |
| FD81 | 12 | F | NR | NR | NR | N | N |
| FD82 | 05 | M | NR | NR | NR | N | N |
| FD83 | 37 | M | NR | NR | NR | N | N |
| FD84 | 03 | M | NR | NR | NR | **NT** | **NT** |
| FD85 | 36 | M | NR | NR | NR | N | N |
| FD86 | 26 | F | NR | NR | NR | N | N |
| FD87 | 44 | M | NR | NR | NR | N | N |
| FD88 | 78 | F | NR | NR | NR | N | N |
| FD89 | 34 | F | NR | NR | NR | N | N |
| FD90 | 38 | M | NR | NR | NR | N | N |
| FD91 | 43 | F | NR | NR | NR | N | N |
| FD92 | 83 | F | NR | NR | NR | N | N |
| FD93 | 63 | F | NR | NR | NR | N | N |
| FD94 | 06 | M | NR | NR | NR | N | N |
| FD95 | 18 | M | NR | NR | NR | N | N |
| FD96 | 10 | F | NR | NR | NR | N | N |
| FD97 | 32 | F | NR | NR | NR | N | N |
| FD98 | 11 | M | NR | NR | NR | N | N |
| FD99 | 68 | F | NR | NR | NR | N | N |
| FD100 | 15 | F | NR | NR | NR | N | N |
| FD101 | 13 | M | NR | NR | NR | N | N |
| FD102 | 53 | M | NR | NR | NR | N | N |
| FD103 | 55 | M | NR | NR | NR | N | N |
| FD104 | 61 | M | NR | NR | NR | N | N |
| FD105 | 17 | M | NR | NR | NR | N | N |
| FD106 | 20 | F | NR | NR | NR | N | N |
| FD107 | 31 | F | NR | NR | NR | N | N |
| FD108 | 33 | M | NR | NR | NR | N | N |
| FD109 | 33 | F | NR | NR | NR | N | N |
| FD110 | 23 | M | NR | NR | NR | N | N |
| FD111 | 24 | F | NR | NR | NR | N | N |
| FD112 | 08 | F | NR | NR | NR | N | N |
| FD113 | 07 | F | NR | NR | NR | N | N |
| FD114 | 03 | M | NR | NR | NR | N | N |
| FD115 | 55 | M | NR | NR | NR | **NT** | **NT** |
| FD116 | 34 | M | NR | NR | NR | N | N |
| FD117 | 48 | F | NR | NR | NR | N | N |
| FD118 | 24 | F | NR | NR | NR | N | N |
| FD119 | 09 | M | NR | NR | NR | N | N |
| FD120 | 11 | M | NR | NR | NR | N | N |
| FD121 | 14 | F | NR | NR | NR | N | N |
| FD122 | 20 | M | NR | NR | NR | N | N |
| FD123 | 05 | F | NR | NR | NR | N | N |
| FD124 | 47 | M | NR | NR | NR | N | N |
| FD125 | 44 | M | NR | NR | NR | N | N |
| FD126 | 79 | F | NR | NR | NR | N | N |
| FD127 | 29 | F | NR | NR | NR | N | N |
| FD128 | 11 | M | NR | NR | NR | N | N |
| FD129 | 08 | F | NR | NR | NR | N | N |
| FD130 | 40 | M | NR | NR | NR | N | N |
| FD131 | 36 | M | NR | NR | NR | **NT** | **NT** |
| FD132 | 22 | M | NR | NR | NR | N | N |
| FD133 | 30 | F | NR | NR | NR | N | N |
| FD134 | 24 | F | NR | NR | NR | N | N |
| FD135 | 61 | F | NR | NR | NR | N | N |
| FD136 | 10 | M | NR | NR | NR | N | N |
| FD137 | 20 | M | NR | NR | NR | N | N |
| FD138 | 09 | M | NR | NR | NR | N | N |
| FD139 | 08 | M | **NT** | **NT** | **NT** | **NT** | **NT** |
| FD140 | 03 | F | NR | NR | NR | N | N |
| FD141 | 24 | F | NR | NR | NR | N | N |
| FD142 | 12 | F | NR | NR | NR | N | N |
| FD143 | 02 | F | NR | NR | NR | N | N |
| FD144 | 17 | F | NR | NR | NR | N | N |
| FD145 | 07 | F | NR | NR | NR | N | N |
| FD146 | 13 | M | NR | NR | NR | N | N |
| FD147 | 18 | F | NR | NR | NR | N | N |
| FD148 | 54 | F | NR | NR | NR | N | N |
| FD149 | 14 | M | NR | NR | NR | N | N |
| FD150 | 05 | F | NR | NR | NR | N | N |
| FD151 | 20 | M | NR | NR | NR | N | N |
| FD152 | 15 | F | NR | NR | NR | N | N |
| FD153 | 25 | F | NR | NR | NR | N | N |
| FD154 | 32 | F | NR | NR | NR | N | N |
| FD155 | 32 | F | NR | NR | NR | N | N |
| FD156 | 10 | M | NR | NR | NR | N | N |
| FD157 | 29 | M | NR | NR | NR | N | N |
| FD158 | 48 | M | NR | NR | NR | N | N |
| FD159 | 56 | F | NR | NR | NR | N | N |
| FD160 | 2 | F | NR | NR | NR | N | N |
| FD161 | 26 | M | NR | NR | NR | N | N |
| FD162 | 44 | M | NR | NR | NR | N | N |
| FD163 | 42 | M | NR | NR | NR | N | N |
| FD164 | 48 | F | **NT** | **NT** | **NT** | **NT** | **NT** |
| FD165 | 57 | M | NR | NR | NR | N | N |
| FD166 | 22 | M | NR | NR | NR | N | N |
| FD167 | 41 | F | NR | NR | NR | N | N |
| FD168 | 58 | M | NR | NR | NR | N | N |
| FD169 | 42 | M | NR | NR | NR | N | N |
| FD170 | 54 | M | NR | NR | NR | N | N |
| FD171 | 53 | M | NR | NR | NR | N | N |
| FD172 | 48 | F | NR | NR | NR | **NT** | **NT** |
| FD173 | 06 | F | NR | NR | NR | **NT** | **NT** |
| FD174 | 34 | M | NR | NR | NR | **NT** | **NT** |
| FD175 | 32 | F | NR | NR | NR | N | N |

**Legend**: IIF: Indirect Immunofluorescense test; CMIA: Chemiluminescent Microparticle Immunoassay; ELISA: Enzyme-Linked Immunosorbent assay; PCR KDNA: Polymerase Chain Reaction KDNA; NPCR sat DNA: Nested Polymerase Chain Reaction satellite DNA; R: reactive; NR: No reactive; P: positive; N: negative; NT: no tested.
