# Supplementary material for: Serological and molecular inquiry of Chagas disease in an Afro-descendant settlement in Mato Grosso do Sul State, Brazil
Source: PLoS One. 2018 Jan 9;13(1):e0189448. doi: 10.1371/journal.pone.0189448 (PMC5760030; doi:10.1371/journal.pone.0189448)
Supplement: S1 Questionnaire — (DOCX) [file pone.0189448.s002.docx]

**S2 Social and Epidemiological questionnaire for Chagas disease (Translated)**

**Fundação Oswaldo Cruz - Technical Office Fiocruz Mato Grosso do Sul**

**Research:** Evaluation of the presence of *Trypanosoma cruzi* in the human population of the Furnas do Dionísio quilombola settlement.

**Primary Investigator**: Gláucia Elisete Barbosa Marcon, Ph.D.

Code:______________

1. Age:_________ Date of birth:_______________

2. In which city or community were you born?

( ) Furnas do Dionísio ( ) Another city in Mato Grosso do Sul_______________________

( ) another Brazilian state __________________

3. Sex

( ) female ( ) male

4. How do you describe your color or race?

( ) Black ( ) White ( ) Indigenous

( ) Brown ( ) Yellow

5. What is your schooling level?

( ) None completed ( ) Primary school

( ) Secondary school ( ) University or higher education

6. Do you know what Chagas disease is?

( ) Yes ( ) No

7. Does anyone in your family have Chagas disease or had it in the past?

( ) Yes ( ) No

8. Are you familiar with the insect that transmits Chagas disease?

( ) Yes ( ) No

9. Have you had physical contact with the insect that transmits Chagas disease?

( ) Yes ( ) No

10. Have you ever had a blood transfusion? If yes, in which year?

( ) Yes year___________ ( ) No

11. Do you know if you tested positive or negative for Chagas disease?

( ) Yes ( ) No

12. Do you feel tired even with minimal exertion?

( ) Yes ( ) No

13. Do you find it difficult to swallow or evacuate?

( ) Yes ( ) No
